# Proton radiotherapy for pediatric tumors: review of first clinical results

**DOI:** 10.1186/s13052-014-0074-6

**Published:** 2014-09-26

**Authors:** Barbara Rombi, Sabina Vennarini, Lorenzo Vinante, Daniele Ravanelli, Maurizio Amichetti

**Affiliations:** Unità Operativa di Protonterapia, Azienda Provinciale per i Servizi Sanitari (APSS), Trento, Italy; Dipartimento di Medicina, Università di Padova, Padova, Italy

**Keywords:** Proton radiotherapy, Pediatric tumors, Late effects, Secondary tumors

## Abstract

Radiation therapy is a part of multidisciplinary management of several childhood cancers. Proton therapy is a new method of irradiation, which uses protons instead of photons. Proton radiation has been used safely and effectively for medulloblastoma, primitive neuro-ectodermal tumors, craniopharyngioma, ependymoma, germ cell intracranial tumors, low-grade glioma, retinoblastoma, rhabdomyosarcoma and other soft tissue sarcomas, Ewing’s sarcoma and other bone sarcomas. Moreover, other possible applications are emerging, in particular for lymphoma and neuroblastoma. Although both photon and proton techniques allow similar target volume coverage, the main advantage of proton radiation therapy is to sparing of intermediate-to-low-dose to healthy tissues. This characteristic could translate into clinical reduction of side effects, including a lower risk for secondary cancers. The following review presents the state of the art of proton therapy in the treatment of pediatric malignancies.

## Review

### Introduction

Recent therapy progress has improved life expectancy in pediatric cancer patients, with a 5-year overall survival (OS) that increased from 39% in 1960 higher than 80% in 2004 [1]. Radiotherapy (RT) is a fundamental part of the multimodality treatment applied to achieve local (LC) and regional control in solid malignancies. Unfortunately, as survivors live longer, they are at risk of experiencing late effects from their treatments, including radiation. For this reason, several combined approaches with chemotherapy and surgery have been used to avoid or reduce RT. Despite of this, many children require radiation and remain at high risk for developing a multitude of serious long-term sequelae that result in psychological – social problems and reduce the quality of life of survivors [2].

**Figure 1:**
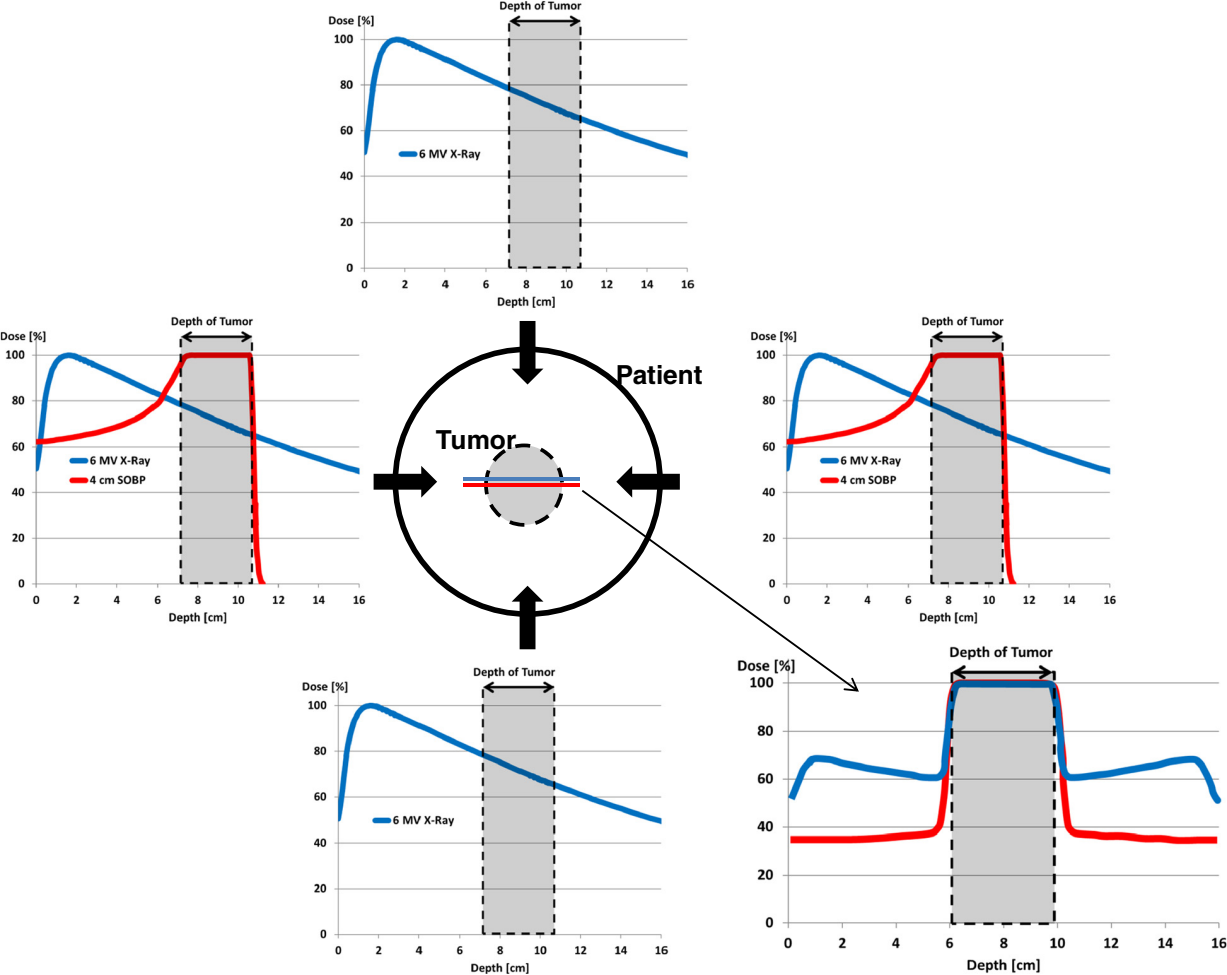
**Simplistic example of depth-dose profiles (PDD) for a 10 cm seated tumor (gray area) within the patient (black circle) using four-fields photon beams and two-fields proton beams (blue and red lines, respectively).** In the bottom right, final PDD due to the sum of individual photon (blue) and proton (red) beams: protons allow a dose reduction of tissues located before and behind the tumor in comparison to photons with the same target coverage.

Protontherapy (PT) is an external RT modality that uses protons instead of photons. These charged particles are accelerated by a cyclotron or synchrotron to reach high energies. When a proton beam enters the body, it delivers a constant dose all through the end of the range where all the remaining energy is deposited within a few millimeters (Bragg peak). The energy and the intensity of the beam can be varied to obtain a longitudinal translation (SOBP: spread–out Bragg peak) and cover the entire target volume. In comparison to high-energy photon treatment, the potential advantages of protons include distal dose fall off, without dose beyond the end of the range, reduction of the dose proximal to the target and reduction of the integral dose (Figure 1). Biologically, protons have not demonstrated a significant advantage over photons, with a relative biological effectiveness of protons of 1.1 compared to photons (1Gy radiobiological equivalent (RBE) = 1Gy × 1.1) [3]. Therefore, tumor control is predicted to be the same for both techniques, but the physical properties of protons allow a better sparing of normal tissues, with the consequent reduction of acute and late toxicities. This ability is particularly important during pediatric age, when the growing organs are very sensitive to radiation effects.

The long life expectancy of survivors and the presence of genetic mutations that induce malignancies (e.g. the RB gene mutation in retinoblastoma) are two factors that cause a significant risk for secondary cancers. The incidence of radiation-induced tumors expected to be reduced using PT, but longer follow-ups are needed for a clinical evidence [4-6].

In a recent review [7], Merchant pointed out some controversial aspects regarding the clinical use of proton therapy for pediatric tumors: the limited number of proton centers which are not geographically well-distributed in the United States country, the lack of proton treatment availability in a timely way, the radiobiological uncertainties of protons might mean substantial differences in dose-volume histogram for proton and photon and that require a better understanding of the radiobiological differences between the two modalities. He concluded that the real benefit of proton over photon therapy based on toxicity reduction would only be realized once proton therapy data will be available for comparison with best existing photon outcome data.

For a definitive spread of this radiation modality, the benefits of protons should be evaluated by results from clinical trials. To date, nearly all-exiting studies on Children’s Oncology Group (COG) for pediatric sarcoma including RMS, CNS tumors, Neuroblastoma, allow proton therapy as radiation treatment modality. In other countries, PT is not always a recognized as integral component of clinical trials. Up to now, although the clinical use of protons has grown, the proportion of patients treated by protons remains still small. One of the reasons is that many proton accelerators were not hospital-based, and were unable to accommodate children that required daily anesthesia and a proton treatment in a timely manner. Another aspect is that the allocation of proton treatment slots in US depends mostly on insurance status. The patients who have proton treatments reimbursed by insurance are significantly smaller than the population who should receive PBT [8].

There are also some physical aspects to consider: as proton technology is still evolving, PT facilities face many issues regarding the treatment planning and targeting of protons. Some of them are: to predict dose deposition due to range uncertainties by modern treatment planning system, the impact of anatomical changing tissue density during proton treatment, breathing, uncertainty regarding RBE [9]. Concern has also been raised about introducing this expensive technology into medical practice and the potential impact on the cost of health care. It is worth noting that while the initial cost of treatment with protons is higher than that of photon therapy, reduced side effects resulting an overall cost savings over a lifetime.

Currently, PT is used in the treatment of intracranial tumors such as ependymoma, medulloblastoma, primitive neuro-ectodermal tumors (PNET), craniopharyngioma, germinoma, and low-grade glioma. Regarding extracranial malignancies, PT is used in case of retinoblastoma, soft-tissue and bone sarcomas of head and neck and paraspinal or pelvic regions. The first clinical results of PT in pediatric oncology are described in the next section.

### First clinical results

#### Ependymoma

The current therapeutic approach for localized ependymoma is the maximal tumor resection followed by RT. A dosimetric comparison study [10] between PT and intensity modulated photon radiation therapy (IMRT) showed a significant dose reduction from radiation of cochlea, temporal lobes, whole brain and hypothalamus with PT, while target coverage and dose to organs close to the target (e.g. brainstem) were similar. An additional sparing of healthy tissues would be possibly obtainable with intensity modulated PT (IMPT).

Recently, MacDonald et al. [11] reported the outcome of 70 patients with localized ependymoma (73% infratentorial, 27% supratentorial) after total or subtotal resection followed by PT. At a median follow-up of 46 months, 3-year LC, progression-free survival (PFS) and OS were 83%, 76% and 95%, respectively. Subtotal resection was significantly associated with worse PFS (54% vs. 88%, p = 0.001) and OS (90% vs.97%, p = 0.001). Only three patients had pituitary gland deficiency after PT (growth hormone deficiency and hypothyroidism) and two patients had hearing loss. Regarding potential neurocognitive sequelae, mean intelligence and adaptive skills after PT were stable in comparison to the baseline evaluation and did not changed in different age groups. No cases of secondary malignancies were identified.

Amsbaugh et al. [12] published the clinical results of 8 cases of spinal ependymoma that received PT after local recurrence (3 cases) or as part of their primary treatment (5 cases). The mean prescribed dose was 51.1 Gy(RBE). After a mean follow-up of 26 months LC, PFS and OS were 100%. There were no events of high-grade toxicity. In comparison to photon therapy, the dose to the Organs At Risk (OARs) anterior to the vertebral bodies (i.e. thyroid, heart, small bowel, stomach) was dramatically reduced.

In conclusion, both dosimetric studies and the first clinical results suggest that treating ependymoma with PT can better preserve hearing, neuro-endocrine and neurocognitive functioning, with similar rates of tumor control obtained by photon RT.

#### Craniopharyngioma

**Figure 2:**
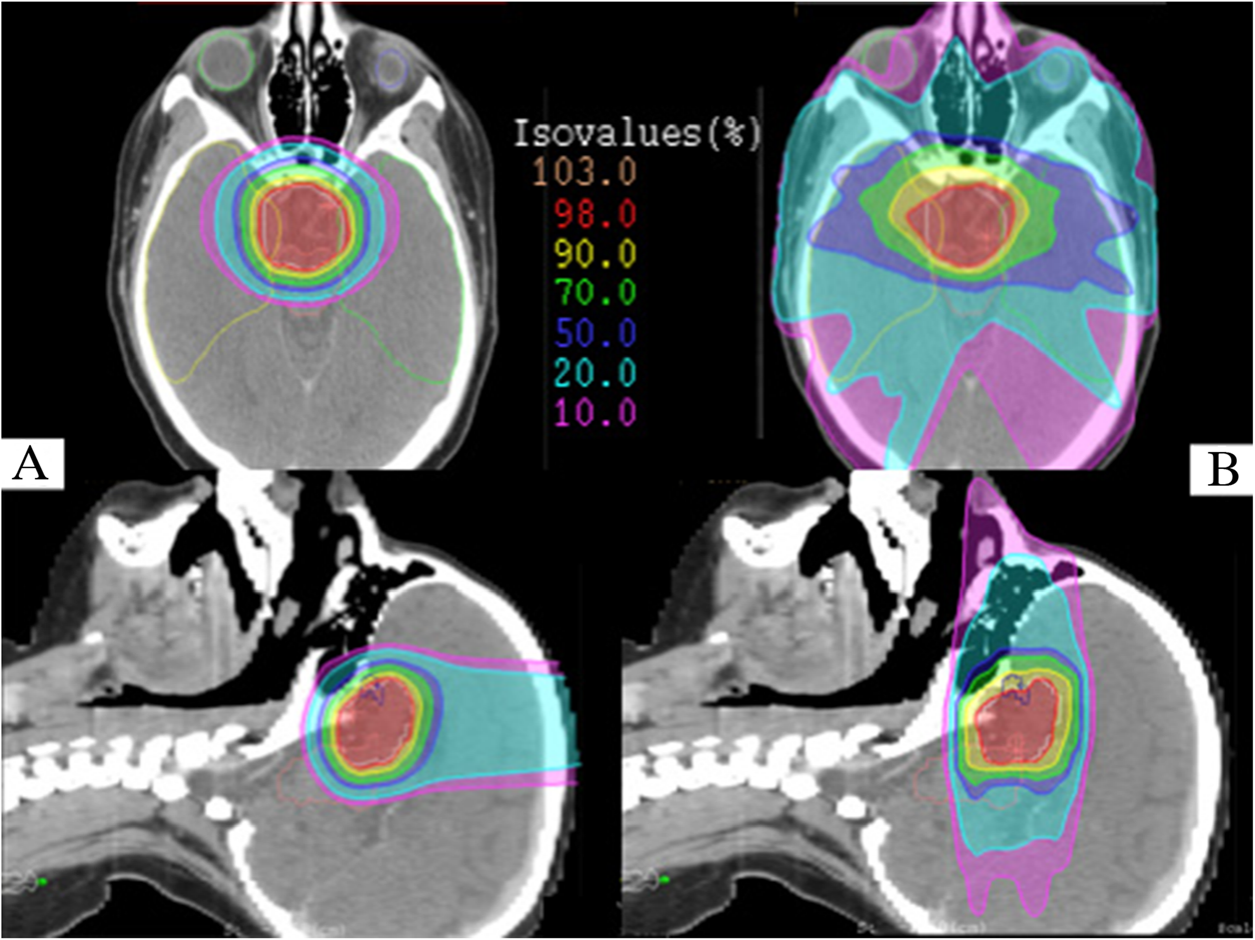
**Axial and sagittal isodose distributions comparing intensity-modulated proton therapy (IMPT) (A) and intensity-modulated radiotherapy (IMRT) (B).** For IMPT plan 3 beam angles were used (1 vertex, 2 symmetric lateral off-axis vertex). For IMRT plan 7 equidistant and coplanar beams were used. PTV (white), right temporal lobe (yellow), left temporal lobe (green), brainstem (pink), left eye (light blue), right eye (light green), chiasma (blue) are outlined. The IMPT plan improved sparing of the temporal lobes, orbital structures and both optic nerves. The integral dose to the brain tissue is decreased with IMPT.

RT is usually delivered after limited surgery, at the time of first diagnosis or at progression, or in exclusive RT setting [13]. The combined approach with limited surgery and irradiation reduces the late toxicity of OARs placed in the chiasmatic or pre-chiasmatic region (i.e. optic chiasm, hippocampi, hypothalamus and pituitary gland) in comparison to radical surgery alone [14]. Merchant et al. [15] evaluated 3 dimensional imaging and treatment-planning data including targeted tumor and normal tissues volumes (entire brain, temporal lobes, cochleas, hypothalamus) of 10 craniopharyngioma patients. Dose-volume data were compared based on proton and photon treatment modality using dose-cognitive effects models. Craniopharyngioma target volume coverage was similar for both treatment modalities. The differences between proton and photon dosimetry showed an advantageous sparing of cochleae, hypothalamus, and normal tissue volumes such as supratentorial brain or temporal lobes, which received less of the low and intermediate doses. Those differences when applied to longitudinal models of radiation dose-cognitive effects resulted in higher IQ scores for craniopharyngioma patients. Figure 2 shows an example of dosimetric comparison between PT and IMRT plans for craniopharyngioma case.

Boehling et al. [16] compared three-dimensional conformal PT (3D-CPT), IMPT and IMRT plans of 10 pediatric patients affected by craniopharyngioma. The target volume coverage was adequate for all modalities but 3D-CPT and even more IMPT reduced the integral dose to hippocampus, dentate gyrus, subventricular zone, brain tissue, cerebellum, brainstem and major cerebral arteries. Similar results were obtained by Beltran et al. [17] who compared the same techniques in 14 craniopharyngioma cases. The best conformal technique was IMPT, which has high sensitivity to target volume changes.

Preliminary clinical results of 16 craniopharyngioma patients treated with post-operative PT to a total dose of 50.4 – 59.4 Gy(RBE) were reported by Luu et al. [18]. After a mean follow-up of 62 months, LC and OS were 93% and 80%, respectively, while 75% of patients were free from late toxicity. The effect of combined surgery and PT on quality of life was investigated by Laffond et al. [19] with specific questionnaires administered to 23 families. Depression was registered in 48% of cases, 38% had dysexecutive symptoms and the majority of families felt “very concerned” by the disease.

In summary, PT showed to be indicated as part of a multidisciplinary management with the goal to reduce late sequelae, especially in regards to neurocognitive functioning.

#### Medulloblastoma and primitive neuro-ectodermal tumor (PNET)

The standard therapy for children older than 3 years with medulloblastoma and PNET is maximal safe resection followed by craniospinal irradiation (CSI) in addition to posterior fossa or tumor bed boost and chemotherapy. Patients younger than 3 years, the role of CSI is still controversial due to important side effects. Using photon beams, the exit dose from the spinal field results in irradiation of normal tissues anterior to the spine i.e. thyroid, heart, lungs, bowel, ovaries, breast and uterus. PT is better able to spare these organs because of the distal dose fall-off in the few millimeters anterior to the vertebral bodies.

Zhang et al. [20] evaluated the predicted risks of cardiac toxicities for a 4-year-old boy receiving photon or proton irradiation CSI for medulloblastoma. They calculated the relative risk (RR) values using a linear risk model and the normal tissue complication probability (NTCP) values using Lyman model. The RR values of cardiac toxicity were 1.28 with protons versus 8.39 with photons. The predicted ratios of NTCP (photons/protons) were much less than unit. They concluded that PT CSI carried a lower risk of radiogenic cardiac toxicity compared to photon CSI.

The relative risk of premature ovarian failure (RRPOF) after cranio-spinal proton radiotherapy was investigated by Pérez-Andújar and colleagues [21]. They calculated the equivalent dose delivered to the ovaries of an 11-year old girl from therapeutic and stray radiation and then predicted the percentage of ovarian primordial follicles killed by radiation and used this as a measure of RRPOF among the three radiotherapies. Proton radiotherapy had a lower RRPOF than conventional photon radiotherapy or IMRT for all sets of ovaries, regardless of uncertainties in ovarian location.

Clair et al. [22] generated three plans of a patient affected by medulloblastoma, while comparing conventional x-rays, IMRT and PT. PT resulted to be the best technique to reduce the dose to structures located beyond the vertebral body and to the cochlea, pituitary, hypothalamus, temporo-mandibular joint, parotid and pharynx. The dosimetric advantage of protons over photons RT was documented by Lin et al. [23] in 9 patients who underwent posterior fossa irradiation. They reported better sparing of cochlea and temporal lobe by PT.

Moeller et al. [24] performed pre- and one-year post-PT audiometric testing in patients treated for medulloblastoma who also received platinum-based chemotherapy. The hearing function of 35 ears was analyzed: the rate of high-grade ototoxicity was low (5%) and the sensitivity to low frequencies was preserved. Jimenez et al. [25] reported the outcomes of 15 very young children (age < 5 years) treated with upfront chemotherapy and subsequent PT (local volume for all patients, with the addition of CSI in 11) for medulloblastoma and PNET tumors. At a median follow-up of 39 months, one patient died for local failure and one from a non-disease related cause. The remaining 13 out of 15 were alive, free of recurrence. 13% patients developed grade 3 ototoxicity requiring hearing aids and 20% had grade 2 neuro-endocrinopathy. The IQ index remained stable during follow-up testing in comparison to baseline evaluation.

The potential reduction of secondary tumors is well documented by radiobiological predictive models [4–6], but clinical data from long term follow-ups are not yet available. This advantage could translate into new clinical indications, as for example the usefulness of breast cancer screening in survivors, considered mandatory after x-ray therapy, that will be unnecessary after CSI with PT [26].

On the basis of dosimetric studies and early clinical outcome reports, PT should be strongly considered when logistically possible for tumors requiring CSI. A recent debate has risen in the radiation oncology community [27,28] about PT as being the only ethically appropriate radiation treatment for medulloblastoma. Hopefully, long-term follow-up will give the answer.

#### Germ cell tumor

RT has a fundamental role for these malignancies that are divided into two histological categories: germinomas and non-germinomatous germ cells tumors (NGGCT). For diffuse germinomas and NGGCT RT is delivered as CSI plus a boost to the initial tumor. For localized germinomas, the target volume is limited to the whole ventricular system followed by a boost to the residual disease [29]. MacDonald et al. [30] compared 3D-CPT and IMPT plans of 22 germinoma patients with IMRT obtaining similar target coverage with all three techniques but a substantial sparing of brain tissue with PT. LC and OS were 100% after a median follow-up of 28 months. The treatment was well tolerated in terms of acute toxicity. Only 4 patients developed late toxicity (2 hypothyroidism and 2 growth hormone deficiency), while no neurocognitive or auditory toxicity were detected. This is currently the only study that shows the early outcome of patients with germ cell tumors treated with PT.

Although preliminary, the results are interesting from both the clinical and the dosimetrical point of view not only for the acute toxicity reduction but also for the potential advantage on late side effects, such as neurocognitive sequelae due to the lower irradiation of the temporal lobes.

#### Low-grade glioma

Surgical resection is the main treatment in low-grade glioma. RT is used particularly for deeply placed tumors, after incomplete surgery or in the definitive setting after post-chemotherapy progression [31]. Hug et al. [32] published the results of 27 patients treated with PT at doses of 50.4-63 Gy(RBE) after a median follow-up of 3.3 years. PT was used as an adjuvant (44%) or salvage (56%) intent obtaining LC and OS rate of 87% and 93% for diencephalic tumors, 71% and 86% for hemispheric tumors, 60% and 60% for tumors placed in the brainstem. All patients with stable disease maintained a good performance status.

In gliomas of the optic nerves, RT is a treatment option that should be considered in case of local progression after surgery or for unresectable tumors. Fuss et al. [33] compared PT plans with 3D-CRT and lateral photon beam techniques and showed similar conformity and homogeneity of dose coverage of the target, but a better sparing of frontal and temporal lobes, contralateral optic nerve and pituitary gland.

RT in low-grade gliomas is associated with possible negative effects in neurocognitive, vascular, visual and endocrine functioning. PT has shown promising results in reducing these adverse events thanks to a better sparing of healthy tissues (i.e. useful vision has been maintained in 57% of patients and improved in 29% as reported by Fuss et al. [33]) while similar local control was obtained.

#### Soft tissue Sarcoma (STS)

RT is usually part of the multimodal approach of STS together with chemotherapy and surgery. PT is usually employed to reduce side effects in cases where the tumor is in close proximity to critical structures. Timmermann et al. [34] treated 16 sarcomas located in different sites, with a total dose of 50.4 - 61.2 Gy(RBE). After a median follow-up of 18.6 months, two-year PFS and OS were 71.6% and 69.3%, respectively; four patients had local failure. PT was well tolerated.

#### Rhabdomyosarcoma (RMS)

Yock et al. [35] compared 3D photon and proton plans of seven patients with orbital RMS treated with PT and evaluated the rate of clinical late effects. PT showed dosimetric advantage limiting the dose to the brain, pituitary grand, hypothalamus, temporal lobes and ipsi/contralateral orbital structures. After a median follow up of 6.3 years, only one local recurrence had occurred. Late toxicity was limited, excellent vision was maintained. Two patients requiring eye drops and none of patients developed neuroendocrine dysfunction. All patients had facial dimorphism, 29% mild bone asymmetry and 71% enophthalmos. Kozak et al. [36] compared PT and IMRT plans of 10 parameningeal RMS patients. PT reduced dose to orbital structures, optic pathways, whole brain temporal lobes, brainstem, pituitary, hypothalamus, parotid and lacrimal glands with similar target volume coverage.

Childs et al. [37] published the clinical outcome of 17 patients with parameningeal RMS treated with PT to a dose ranged between 50.4 and 56 Gy (RBE). With a median follow-up of 50 months, the 5-year PFS and 5-year PFS were 59% and 64%, respectively, comparable with historical photon cohorts. Late effects included failure to maintain height velocity (n = 3), endocrine deficits (n = 2), mild facial hypoplasia (n = 7), failure of tooth eruption (n = 3), dental caries (n = 5) and chronic nasal/sinus congestion (n = 2). Functional results were better in comparison to those observed in photon studies [38,39].

Cotter et al. [40] compared IMRT and PT plans for 7 patients with bladder/prostate RMS. PT led to a significant decrease in mean dose to bladder, testes, femoral heads, growth plates and pelvic bones compared to IMRT. After a median follow-up of 27 months, two recurrences were observed. Late effects related to PT were very limited: only one patient had intermittent hematuria.

#### Ewing sarcoma

In the multimodality management of Ewing sarcoma, RT is usually used in post-operative setting for patients with close or positive resection margins after poor response post-chemo or as a definitive intent for unresectable tumors. Rombi et al. [41] published initial clinical outcome of 30 Ewing’s sarcoma patients treated with PT at a median prescribed dose of 54 Gy(RBE). At a median follow-up of 38.4 months, 3-year LC, PFS, and OS were 86%, 60%, and 89%, respectively. The PT was very well tolerated and acute Grade 3 side effects occurred in 6 cases (5 skin desquamation and 1 fatigue). Five patients (16.7%) who received surgical laminectomy prior to radiation developed scoliosis/kyphosis (three mild, one moderate, one severe) and 20% had permanent skin changes. Four patients developed secondary hematological tumors (three acute myeloid leukemia and one myelodysplastic syndrome), potentially due to high doses of alkylating antineoplastic agents.

In conclusion, PT may be indicated in selected cases of STS, RMS and Ewing sarcoma, especially in orbital, parameningeal, paraspinal and pelvic sites to mitigate late toxicity. Further investigations to evaluate the impact of PT on bone impairment are needed.

#### Chordoma and Chondrosarcoma

In chordoma and chondrosarcoma RT may be prescribed in post-operative setting or with exclusive intent. It is usually delivered at high doses up to 70–76 Gy(RBE). These tumors are always in close proximity to critical structures, as brainstem, optic pathways, brain tissue and spinal cord. Chordomas and chondrosarcomas are historically the typical pathologies referred to PT facilities. Habrand et al. [42] evaluated clinical results of 30 children treated with combined photon and proton RT, with a mean follow up of 26.5 months. Five year PFS was 100% for chondrosarcoma and 77% for chordoma and 5-year OS was 100% for chondrosarcoma and 81% for chordoma. The acute tolerance to PT treatment was very good; only one late severe toxicity case (hearing loss) was registered. Rombi et al. [43] reported the outcome of 26 patients treated with PT after a mean follow up of 46 months. In chordoma cohort, the 5-year LC and 5-year OS were 81% and 89%, respectively; in chondrosarcoma cohort, the 5-year LC and 5-year OS were 80% and 75%, respectively. Relatively few (19%) late complications were described, with no events of severe grade.

PT is considered a useful RT modality for chordoma and chondrosarcoma due to the capability to escalate the dose with acceptable rates of late toxicity.

#### Retinoblastoma

RT is commonly used to preserve the eye and the visual function in advanced retinoblastomas or in the post-operative setting for high-risk tumors. Krengli et al. [44] showed that PT is useful to reduce the integral dose to healthy tissue, decreasing the risk of bone impairment and functional sequelae. Also, secondary malignancies could be reduced particularly in children with hereditary RB gene mutation, associated with a higher risk of radiation-induced tumors. Sethi et al. [45] have recently compared the risk for secondary malignancies in patients with retinoblastoma who were treated with PT (55 patients) and photon radiotherapy (31 patients). The median follow up was 6.9 years in proton cohort and 13.1 years in photon cohort. Ten-year cumulative incidence of radiation-induced second malignancies was 0% vs.14% (p = 0.015), respectively.

PT Department at Massachusetts General Hospital of Boston is treating patients with advanced or high-risk retinoblastoma with preliminary good clinical results in regards to secondary-tumor reduction, functional and cosmetic outcomes [45].

#### Neuroblastoma

RT is currently part of treatment for high-risk neuroblastoma and it is usually delivered on primary tumor site with limited doses after neoadjuvant chemotherapy and surgery. The role of PT in neuroblastoma is mainly investigated for the retroperitoneal site, which accounts for about the 40% of all cases. Hattangadi et al. [46] compared dosimetrically 3 different plans (IMRT, 3D-CPT, and IMPT) of 9 patients. The dosimetric advantage of 3D-CPT over IMRT was demonstrated in all cases, while IMPT allowed additional sparing of kidneys, lungs and heart. In this paper, all cases were treated with 3D-CPT and at a median follow up of 38 months, and no local failures were detected. Few acute and late side effects were detected without high-grade toxicity.

Hill Kayser et al. [47] compared 3D-CPT with IMRT of 13 patients in order to choose the most appropriate technique in relation to tumor location. For 9 patients with lateralized disease, PT offered sparing of the contralateral kidney but in two cases, IMRT improved overall bilateral renal sparing. They concluded that PT offered optimal combination between target coverage and OARs sparing (i.e. bowel, both kidneys, liver, heart, lungs) in most of the cases (11/13) with 100% of local control occurred after a mean follow up of 16 months.

Fuji et at. [48] compared 3D-CPT, 3D-CRT and IMRT plans of 5 cases and obtained similar results regarding dose sparing. Additionally, by using 3D-CPT the secondary cancer risk calculated using a model, which took in account the rates of cell killing, repopulation and neutron dose from treatment, was lower in all organs evaluated (i.e. liver, stomach, small intestine, colon, bone) except in the pancreas.

The dosimetric evaluations and the first clinical results of PT in high-risk neuroblastoma are promising. The potentiality of PT to reduce the dose to organs outside the target volume could improve the tolerance of a very aggressive multimodal treatment and decrease the incidence of secondary cancer.

#### Lymphoma

In recent years, numerous comparative studies have been conducted to evaluate the potential role of PT in mediastinal lymphoma. Hoppe et al. [49] evaluated the plans of stage II-III Hodgkin’s lymphoma (HL) cases with mediastinal location and they confirmed the advantage of 3D-CPT over conformal RT and IMRT to reduce the dose to the heart, lungs, esophagus, thyroid and breast. The same authors [50] published a second article mainly focused on heart structures, in particular the coronary arteries, with the potential reduction of late ischemic damage by PT use. Andolino et al. [51] found that using 3D-CPT in pediatric female patients with stage II-HL the dose to the breast was reduced of 80%.

In dosimetric comparison studies of protons and photons in several tumor types and locations, PT appears promising in reducing the dose to OARs; it could translate into reduced incidence of secondary cancer (especially breast cancer) but clinical evidence are still limited.

## Conclusions

RT is effective in increasing local control in several pediatric tumors, but it is often associated with severe late effects, including secondary tumors. The physical advantages of protons, which decrease the dose to healthy tissues, are promising in achieving significant clinical benefits. Dosimetric comparison studies pointed out the superiority of protons over photons in several tumor locations. Clinical data are still limited, but the first evidence generally confirmed an outcome similar to historic photon cohort, but better tolerance to PT and few side effects, that could have positive impact also on the survivors’ quality of life.

Several proton facilities have started their activity during the last decade and new centers are planned worldwide. The spread of the PT centers in the near future will make this technology easily accessible to pediatric patients. With the increase of follow-up time, the evidence in favor of PT could become more solid. Specialized teams of radiation oncologists, medical physicists, anesthesiologists, psychologists and nurses are needed and new observational protocols should be designed to investigate the benefits on late toxicity and quality of life.

## Abbreviations

RT: Radiotherapy
PT: Protontherapy
IMRT: Intensity modulated radiotherapy
IMPT: Intensity modulated protontherapy
3D-CPT: Three dimensional conformal proton therapy
3D-CRT: Three dimensional conformal radiotherapy
RBE: Radiobiological equivalent
OAR: Organ at risk
OS: Overall survival
LC: Local control
PFS: Progression-free survival
CSI: Craniospinal irradiation
PNET: Primitive neuro-ectodermal tumors
NGGCT: Non-germinomatous germ cells tumors
HL: Hodgkin’s lymphoma
